# The B-Health Box: A Standards-Based Fog IoT Gateway for Interoperable Health and Wellbeing Data Collection

**DOI:** 10.3390/s25237116

**Published:** 2025-11-21

**Authors:** Maria Marques, Vasco Delgado-Gomes, Fábio Januário, Carlos Lopes, Ricardo Jardim-Goncalves, Carlos Agostinho

**Affiliations:** 1 Center of Technology and Systems (UNINOVA-CTS), Associated Lab of Intelligent Systems (LASI) and Confederation of Laboratories for Artificial Intelligence Research in Europe (CAIRNE), FCT Campus, 2829-516 Caparica, Portugal; vmdg@uninova.pt (V.D.-G.); faj@uninova.pt (F.J.); csl@uninova.pt (C.L.); rg@uninova.pt (R.J.-G.); ca@uninova.pt (C.A.); 2 IDEA Institute, Centro Cultural e de Investigação do Funchal, 9000-100 Funchal, Portugal; 3 Department of Electrical and Computer Engineering, NOVA School of Science and Technology (FCT NOVA), 2829-516 Caparica, Portugal

**Keywords:** IoT, fog computing, digital health, EHR, HIS, FHIR, interoperability, wearables, workplace

## Abstract

In recent years, healthcare is evolving to meet the needs of a growing and ageing population. To support better and more reliable care, a comprehensive and up-to-date Personal Health Record (PHR) is essential. Ideally, the PHR should contain all health-related information about an individual and be available for sharing with healthcare institutions. However, due to interoperability issues of the medical and fitness devices, most of the times, the PHR only contains the same information as the patient Electronic Health Record (EHR). This results in lack of health-related information (e.g., physical activity, working patterns) essential to address medical conditions, support prescriptions, and treatment follow-up. This paper introduces the B-Health IoT Box, a fog IoT computing framework for eHealth interoperability and data collection that enables seamless, secure integration of health and contextual data into interoperable health records. The system was deployed in real-world settings involving over 4500 users, successfully collecting and transmitting more than 1.5 million datasets. The validation shown that data was collected, harmonized, and properly stored in different eHealth platforms, enriching data from personal EHR with mobile and wearable sensors data. The solution supports real-time and near real-time data collection, fast prototyping, and secure cloud integration, offering a modular, standards-compliant gateway for digital health ecosystems. The health and health-related data is available in FHIR format enabling interoperable eHealth ecosystems, and better equality of access to health and care services.

## 1. Introduction

The healthcare sector is undergoing significant transformation in response to demographic shifts, particularly the rising number of older adults and individuals with chronic conditions. This evolution is driving a shift toward more proactive, personalized, and data-driven models of care. As new technologies and medical innovations become available, healthcare systems must adapt to deliver more timely, efficient, and patient-focused services.

One central element for this to happen is the existence of up-to-date Electronic Health Records (EHR) to systematize the collection of electronically stored patient and population health information in a digital format. However, and while EHR standardizes health data, it does not determine or specify which standards to adopt, and the EHR integration process in health information systems (HIS) is complex due to the paper-based medical processes that are still being used [1]. Also, currently, patients have only limited information of their Electronic Health Record (EHR), such as, e.g., discharge summary and reports which was a problem in eHealth consultations during the COVID-19 pandemic [2].

To overcome these difficulties, the concept of Personal Health Record (PHR) emerged aiming to empower individuals with their health data in real-time and enable them to share it with trusted persons, in an interoperable and safe manner [3]. The PHR serves as a comprehensive repository of an individual’s health-related data, encompassing medical history, prescriptions, diagnostic results, and lifestyle-related information [4]. By making this data readily available for sharing with healthcare institutions, PHRs play a pivotal role in enhancing healthcare delivery and ensuring continuity of care [5].

ISO 14639-2 standard [6] indicates that PHR is the “representation of information regarding or relevant to the health, including wellness, development, and welfare, of a subject of care, which may be stand-alone or integrating health information from multiple sources” [7]. PHR systems differs from EHR systems in three main respects [3]:

1. PHRs should be under patients’ control, in compliance with EU GDPR.
2. PHRs should include real-time data from patients’ medical monitoring sensors.
3. PHR management systems should support data interoperability.

However, due to interoperability issues and lack of integration of wellbeing and other complementary medical devices, most PHRs today contain the same information as a patient Electronic Health Record (EHR), which primarily focuses on medical diagnoses, treatment plans, and hospital visits. This limited scope results in a lack of crucial health-related information, such as physical activity levels, sleep patterns, and working habits. These factors are increasingly recognized as essential determinants of overall health, influencing disease prevention, treatment efficacy, and long-term patient outcomes [8]. The absence of such comprehensive data within PHRs hinders healthcare providers from making fully informed decisions regarding medical conditions, proper prescription management, and effective treatment follow-up [9]. For example, environmental sensors can provide valuable information to the PHR about where the monitored patient is located, such as the temperature, the level of humidity, the lighting, as well as the level of patient’s sweat which can be measured by advanced smart bed, etc., allowing the medical staff to achieve more accurate diagnosis and thus deliver more efficient treatment [10]. These devices can generate huge amounts of data enabling the possibility to develop data analytics for, e.g., pattern identification for better patient care. However, this is only possible if a standardized approach to data collection is implemented with a focus on data interoperability.

This paper proposes the B-Health IoT Box, a fog computing gateway designed to bridge this interoperability gap by collecting, harmonizing, and transmitting health-related data from heterogeneous sources. To address these issues, innovative Internet of Things (IoT) frameworks for eHealth interoperability and data collection are being developed. The World Health Organization (WHO) defines eHealth as “the transfer of health resources and healthcare by electronic means” [11], highlighting the role of IT-based systems, including Machine-to-Machine (M2M) communication and IoT, in capturing and digitizing real-world health data. In very simple terms, IoT combines consumer products, industrial components and other everyday objects with internet connectivity and powerful data analytical capabilities that can transform the way people work and live [12]. The objective is to optimize the functionalities of each object by transforming every piece of extractable data into actionable insights. When connected to an intelligent network, these objects can adapt to their context and even take on new functions, enabled by real-time analysis and computation [13]. Despite the growing availability of such systems, few solutions have been validated at scale in real-world healthcare deployments. This paper addresses that gap. The capability of integrating data from diverse sources, including wearables, smart sensors, and medical devices, allows for enriching the PHR with a broader spectrum of health-related insights [14]. By leveraging IoT technology, health platforms can harmonize the collected data and ensure its compatibility with established eHealth standards such as HL7 FHIR [15] and ISO/IEEE 11073 standards [16]. This seamless data integration fosters a more holistic approach to patient care, enabling healthcare professionals to incorporate real-time health indicators into their clinical assessments and decision-making processes [17].

Furthermore, the implementation of IoT-driven interoperability solutions has the potential to revolutionize healthcare delivery by facilitating proactive and personalized care. For instance, continuous monitoring of an individual’s physical activity and vital signs can help detect early warning signs of chronic conditions, prompting timely medical interventions [18]. Additionally, data-driven insights derived from PHRs can support precision medicine initiatives, allowing for tailored treatment strategies that align with an individual’s unique health profile [19]. By harnessing IoT frameworks in conjunction with edge and fog computing, Artificial Intelligence (AI) and Machine Learning (ML), big data, blockchain, and cloud computing, healthcare institutions can bridge the existing gaps in health information management and foster a more patient-centric approach to care [20]. As these technologies continue to advance, the seamless integration of diverse health data sources will be instrumental in driving better health outcomes, improving treatment adherence, and ultimately enhancing the overall quality of healthcare services [21].

However, the adoption of IoT infrastructures for healthcare still presents several challenges, particularly when integrated with complex, data-intensive architectures and emerging digital technologies. These challenges include the following:

- Data Management: The vast amount of data generated by IoT devices, coupled with the complexity of integrating edge, fog, and cloud computing, requires advanced data governance models. AI and ML techniques must be employed to analyze real-time health data efficiently while ensuring accuracy and reducing redundancy [18].
- Scalability: As healthcare IoT ecosystems expand, the ability to scale infrastructure without compromising performance is a significant challenge. Cloud computing and fog computing offer solutions by distributing computational loads; however, balancing latency and bandwidth efficiency remains an issue [19].
- Interoperability, Standardization, and Regulatory Affairs: Different medical devices and platforms use various data formats and communication protocols, which complicates seamless data exchange. Blockchain technology is being explored for secure and transparent data transactions, but regulatory compliance (e.g., GDPR, HIPAA) adds another layer of complexity [20,21].
- Interfaces and Human-Factor Engineering: The effectiveness of IoT-based healthcare depends on how easily both patients and medical professionals can interact with these systems. AI-driven natural language processing and intuitive interfaces are being developed to enhance user experience while reducing errors [8].
- Security and Privacy: The increasing volume of sensitive health data collected by IoT devices heightens concerns about security vulnerabilities. Blockchain and AI-driven anomaly detection methods are being leveraged to enhance data security, enforce access control, and prevent cyber threats [14].

To address some of these challenges, this paper proposes the B-Health IoT Box, a fog-based IoT health gateway to tackle eHealth data collection and management as well as interoperability and standardization. The B-Health IoT Box enables the collection and integration of heterogeneous data from different devices (e.g., wearables, smart sensors, medical devices), harmonizing the collected data for further ingestion into health platforms using well established eHealth standards. Section 2 of this paper presents the state-of-the-art of the related technologies and describes the identified gaps. In Section 3 the paper examines the B-Health IoT Box device, its architecture, and the supported eHealth standards. The use case scenarios are presented in Section 4, and validation and discussion are presented in Section 5. Lastly, Section 6 concludes the paper and describes the future work.

## 2. State-of-the-Art

### 2.1. Internet of Things (IoT) and Internet of Medical Things (IoMT)

The Internet of Things (IoT) has transformed industries by enabling connected devices to collect, share, and analyze data [22]. This interconnectivity leads to enhanced automation, efficiency, and decision-making. A specialized subset of IoT, the Internet of Medical Things (IoMT), focuses on healthcare applications, offering solutions for patient monitoring, remote diagnostics, and personalized medicine [23]. These advancements improve healthcare efficiency and patient outcomes by integrating wearables, smart sensors, and AI-driven analytics [24]. However, many of these systems are fragmented, and lack standardized methods for sharing data across devices, platforms, and health systems.

One of the key areas of development in IoT is resource discovery techniques. Efficient resource discovery is crucial for the scalability of IoT systems, as it enables seamless communication and interaction between devices. Researchers have developed AI-driven discovery methods that enhance device interoperability and network efficiency [25]. These advancements make IoT networks more adaptive and responsive, which is particularly beneficial in applications such as smart cities, industrial automation, and environmental monitoring. However, in the context of health, integration of data from diverse sources into a common Personal Health Record remains largely unresolved. Interoperability, latency, security, and regulatory compliance are frequently cited as barriers.

Another significant advancement in IoT is related to security and privacy challenges. As the number of IoT devices increases, so do concerns about cybersecurity. Machine learning-based intrusion detection systems are being developed to identify and mitigate security threats [26]. Blockchain technology is also being integrated into IoT to provide tamper-proof data storage and enhanced authentication protocols [27]. These innovations help protect user data and ensure the integrity of IoT networks.

The integration of IoT with emerging technologies such as 5G networks, edge computing, and AI has further expanded IoT network capabilities. This convergence enables low-latency, real-time data processing, which is critical for applications like autonomous vehicles, industrial IoT, and digital twins [28]. With these advancements, IoT continues to evolve into a more robust and efficient system for managing interconnected devices.

The IoMT market has grown significantly in recent years, driven by the increasing demand for remote healthcare, telemedicine, and AI-driven diagnostics [29]. IoMT devices such as smart insulin pumps and remote ECG monitors enable continuous patient monitoring, leading to improved chronic disease management and overall patient care [30]. As healthcare systems become more reliant on digital tools, the role of IoMT in delivering personalized and efficient healthcare services continues to expand.

One of the most promising developments in IoMT is its role in Healthcare 4.0 [31]. This concept represents the integration of AI, cloud and fog computing, and IoT to create intelligent hospital systems. These smart hospitals leverage real-time data analysis to optimize patient care and resource allocation [32]. The use of AI-driven hospital management systems helps streamline operations, reduce wait times, and improve overall efficiency.

Security remains a major concern in IoMT, as medical data is highly sensitive. To address this challenge, researchers are implementing robust encryption techniques, zero-trust architectures, and compliance frameworks such as GDPR and HIPAA [33]. AI-powered anomaly detection systems are also being deployed to identify and mitigate cyber threats within IoMT networks [34]. These advancements help secure patient data against unauthorized access.

Remote patient monitoring has become a key application for IoMT, enabling continuous tracking of patient vitals, including heart rate, blood pressure, and glucose levels, through wearables and connected medical devices [35]. This continuous monitoring allows healthcare providers to detect health anomalies early and intervene before conditions worsen, also contributing to reduce hospital readmissions and easing the pressure on healthcare systems.

AI-powered health analytics integrated with IoMT is reshaping how patient data is interpreted. By applying ML models to data streams from wearable and connected devices, healthcare providers can analyze patient data more effectively, predict disease progression, personalize treatment strategies, and improve clinical outcomes [36]. Emerging approaches like Federated learning further enhance this potential by enabling decentralized AI model training without compromising patient privacy [37].

While IoMT focuses on improving patient outcomes, predictive maintenance of medical equipment is another significant application of IoT in healthcare. IoT sensors can monitor the performance of medical devices such as MRI scanners and ventilators, providing real-time data on their condition [38]. By analyzing this data, hospitals can anticipate potential failures and perform maintenance before critical issues arise. This proactive approach helps reduce downtime and extend the lifespan of medical equipment.

Nonetheless, despite its advancements, IoT and IoMT still face several challenges that must be addressed to maximize their potential, in particular those associated with interoperability [39]. The success and potential scalability of the IoT strongly depends on enabling heterogeneous devices, networks, and platforms to seamlessly communicate and work together [40]. Without interoperability, IoT ecosystems become fragmented, leading to vendor lock-in, limited functionality, and increased integration costs [41]. As devices from different manufacturers often operate using distinct communication protocols, data formats, and interfaces, standardized interoperability is essential to ensure they can interact in real time and contribute meaningfully to automated processes and decision-making systems [42].

#### 2.1.1. Key Interoperability Standards and Protocols for IoMT Systems

Standard data formats enable semantic interoperability by ensuring that transmitted information maintains consistent structure and meaning across systems, which is vital for downstream processing, analytics, and decision-making [43]. The same applies for communication protocols to dictate how data is transmitted and reliably communicate regardless of manufacturer [44]. Also, the adoption of carefully chosen interfaces enhances plug-and-play compatibility, reduces integration costs, and facilitates device replacement or scaling [45]. In Table 1, an overview of the most commonly used protocols for IoMT interoperability is provided.

##### Continua Design Guidelines (CDG)

The Continua Design Guidelines (CDG), developed by the Personal Connected Health Alliance (PCHAlliance), serve as an essential interoperability framework within the Internet of Medical Things (IoMT) ecosystem. Rather than introducing new protocols, CDG leverages and constrains existing standards, such as IEEE 11073, Bluetooth Health Device Profile (HDP), USB Personal Health Device Class (PHDC), and HL7, to define device communication and end-to-end system interoperability in personal connected health systems [46].

CDG is particularly relevant for home-based and mobile health monitoring, where devices from various manufacturers must interact seamlessly with gateways and backend systems. The CDG architecture outlines three primary components:

- Personal health devices (e.g., glucose metres, pulse oximeters);
- Application hosting devices (e.g., smartphones or hubs that aggregate data);
- Health record systems (e.g., cloud platforms or EHRs).

In practice, for example, a Continua-compliant blood pressure monitor using ISO/IEEE 11073-10407 [47] can transmit data via Bluetooth HDP to a smartphone, which then relays the information using HL7 messaging to a clinical backend. CDG also includes security and privacy recommendations for protecting personal health information during transmission and storage.

A key feature of CDG is its certification process, ensuring that devices adhere to interoperability and functional requirements. Products bearing the Continua certification logo are verified for seamless plug-and-play functionality, which is critical in healthcare environments where usability and reliability directly affect patient outcomes [48].

CDG’s focus on implementation-level interoperability distinguishes it from broader or more abstract standards, making it particularly effective for deployment in real-world IoMT environments [49].

##### Fast Healthcare Interoperability Resources (FHIR)

Fast Healthcare Interoperability Resources (FHIR), developed by HL7, is a next-generation interoperability standard designed to simplify the exchange of health information using modern web technologies such as RESTful APIs, JSON, and XML [50]. FHIR is structured around granular, reusable components called “resources” (e.g., Observation, Device, Patient), which can be combined and extended to represent complex clinical scenarios.

In IoMT applications, FHIR plays a critical role in transforming raw sensor data into machine-readable, semantically structured formats. For example, a wearable ECG device may generate a daily summary of heart rate variability, which is encoded as a FHIR Observation resource and transmitted to a remote patient monitoring platform. This facilitates real-time analytics, decision support, and integration into EHRs [51]

FHIR’s value in IoMT is further amplified through its integration with other standards. For example, FHIR can receive data originating from CDG-compliant devices (which use IEEE 11073 or HL7 v2) by transforming those data streams at the gateway or server level into FHIR resources. Additionally, frameworks such as SMART on FHIR enable secure, user-authenticated access to FHIR data via apps on smartphones and tablets [52].

Despite its strengths, FHIR implementation in resource-constrained IoMT devices can be challenging. In such cases, data is often pre-processed or normalized at the edge (e.g., by a mobile app or hub) before FHIR serialization and transmission to backend systems.

##### Integrating the Healthcare Enterprise (IHE) Profiles

Integrating the Healthcare Enterprise (IHE) is a global initiative that promotes the coordinated use of established standards such as HL7, DICOM, and FHIR to achieve seamless interoperability among healthcare IT systems. In the context of IoMT, IHE provides a suite of integration profiles that specify workflows and technical parameters for incorporating medical devices and remote data sources into clinical environments [53].

Two IHE profiles are particularly relevant to IoMT:

- Patient Care Device (PCD): Facilitates the integration of data from medical devices (e.g., infusion pumps, ventilators, wearable monitors) into clinical systems [54].
- Mobile Care Services Discovery (mCSD): facilitates the creation, updating, deleting and discovery of care service resources using a RESTful interface in interrelated, federated environments [55].

For remote monitoring scenarios, IHE also supports profiles for cross-enterprise document sharing (XDS) [56] ensuring that data from can be queried, retrieved, and acted upon across organizational boundaries [55].

### 2.2. Architectural Approaches to IoT Interoperability

Traditional IoT architectures have primarily been cloud-centric. In this model, IoT devices collect data through sensors and transmit it to the cloud for processing and storage. The cloud layer provides computational resources to analyze data, generate insights, and communicate results back to devices or users [57]. However, this approach has significant drawbacks, including high latency, increased bandwidth costs, and privacy concerns. These challenges have led to the emergence of alternative architectural models such as edge and fog computing, which aim to distribute computational tasks closer to the data source [58].

#### 2.2.1. Edge-Based Approach

Edge IoT architectures integrate computing resources into gateways, routers, and embedded devices, allowing data to be processed locally—at or near the data source—before being transmitted to the cloud. This device-level decentralized approach enhances scalability and ensures that critical applications can operate with minimal latency, even during network disruptions [59]. The adoption of edge-computing approaches is driven by the need for high-speed analytics, reduced dependency on cloud infrastructures, and improved security measures that keep sensitive data closer to its point of origin [60]. By distributing computation across the network edge, these architectures alleviate network congestion and optimize bandwidth usage, which is particularly valuable in environments with limited connectivity, such as remote healthcare facilities. Moreover, edge computing reduces operational costs associated with cloud storage and data transfer, making IoT deployments more cost-effective over time [61].

In the healthcare sector, edge computing plays a crucial role in enabling real-time patient monitoring and immediate response. Wearable and IoMT devices can process health data locally, allowing for immediate alerts in case of critical conditions such as irregular heart rhythms or drops in oxygen levels. This local processing supports more personalized and responsive care while reducing the load on centralized IT infrastructures. For instance, edge-enabled systems have shown value in early detection of emergencies like heart attacks or strokes, and in managing elderly or chronically ill patients, where rapid interventions are often needed [62,63]. Edge devices can rapidly interpret signs, reducing the time for critical decision-making [64,65].

Also, the decentralized nature of edge computing can help in ensuring that sensitive data does not have to travel long distances to be processed, thus reducing exposure to potential breaches [66]. Additionally, integrating blockchain with edge computing in healthcare can further enhance data security and integrity [67]. Edge computing can also be used for efficient resource allocation in healthcare environments, ensuring that critical care is always available when needed and to improve telemedicine by enabling faster processing of patient data, thus facilitating real-time consultations and diagnostics [68,69]. In context of eHealth, edge computing can improve the functionality of virtual assistants that help elderly patients manage their health and interact with healthcare professionals remotely [70].

Despite its advantages, challenges related with limited computational power available on edge devices and interoperability constraints remain a challenge in Edge IoT.

#### 2.2.2. Fog-Based Approach

Fog IoT architectures extend the concept of edge by introducing an intermediate layer between edge devices and the cloud. In fog architectures, computational nodes, known as fog nodes or gateways, are deployed closer to end-users to aggregate, filter, and process data before sending relevant information to the cloud [71]. This model enables enlarged computational power when compared to the edge approach, while preserving distributed processing and scalability, and lower latencies than traditional cloud architectures.

The main features of the fog node include (i) connectivity to facilitate seamless communication between IoT devices, gateways, and cloud systems; (ii) two-way data exchange allowing devices to not only send information but also receive commands, updates, and control signals; (iii) flexibility to integrate heterogeneous devices and protocols; (iv) protocol translation allowing IoT devices operating on various standards (e.g., MQTT, CoAP, OPC UA, HL7 FHIR) to exchange data efficiently; (v) data aggregation, filtering formatting, and encoding/decoding to optimize bandwidth usage and reduce cloud dependencies; (vi) short-time database to temporarily hold data before forwarding it to long-term cloud storage or centralized databases; (vii) security and data protection incorporating advanced cybersecurity measures to protect information, (viii) assessment and notification enabling real-time analytics and event-driven alerts, ensuring that critical insights are generated close to the data source; (ix) and local processing allowing immediate decision-making at the network edge [72].

In most IoT-based healthcare systems, especially at smart homes or hospitals, a bridging point (i.e., gateways) is needed between sensor infrastructure network and the cloud system. These gateways translate between diverse communication protocols, manage local data processing, and maintain situational awareness of both the sensor infrastructure and transmitted data [73]. In smart hospital infrastructures, fog gateways handle data preprocessing from connected medical devices before forwarding only relevant information to cloud-based Electronic Health Record systems. This reduces bandwidth consumption and enhances real-time decision-making capabilities, improves response times, reduces reliance on cloud resources, and improves data security, particularly in critical applications such as smart intensive care units [74,75].

The integration of AI with fog computing has further enhanced healthcare analytics in a way not possible with simple edge-models, enabling real-time predictions and automated decision support. Federated learning techniques have been employed within fog architectures to train machine learning models across distributed healthcare facilities without compromising patient privacy. This approach allows hospitals to develop predictive analytics models for disease detection and progression monitoring while ensuring compliance with data protection regulations such as the General Data Protection Regulation (GDPR) [76]. Another emerging application of fog approaches in healthcare is digital twin technology, where real-time patient data is processed within fog nodes to create virtual simulations of patient health conditions. This approach allows healthcare providers to anticipate disease progression, optimize treatment plans, and conduct personalized medicine interventions [77].

By bridging the gap between edge computing and cloud infrastructures, fog computing stands as a foundational technology in the transformation of healthcare IoT ecosystems [78].

#### 2.2.3. Reference Architectures

As IoT architectures continue to evolve, hybrid models that combine cloud, edge, and fog computing are becoming increasingly prevalent. Hybrid architectures leverage the strengths of each computing paradigm to balance performance, scalability, and cost-effectiveness. For example, in a smart healthcare system, edge computing may handle real-time patient monitoring, fog computing can process aggregated data at hospital gateways, and cloud computing can store historical health records for in-depth analytics and AI-driven diagnostics [79]. This layered approach ensures optimized resource utilization and enhances system resilience against network failures [80].

To support the development of most effective IoT architectures, standardization organizations and initiatives have developed reference architectures to provide best practices, guidelines, and standardized patterns for their design. One example is the IoT World Forum (IoTWF) Reference Model [81], a widely recognized framework that provides a structured approach to designing and implementing IoT architectures. Developed by the Internet of Things World Forum (IoTWF), this model defines a seven-layered architecture that facilitates interoperability, security, scalability, and efficient data flow across IoT ecosystems: the Perception Layer (Physical Devices and Controllers), the Network Layer (communication protocols), the Edge Layer (Edge Computing), the Data Abstraction (data normalization, filtering, and standardization), the Application Layer (software applications), the Collaboration and Processes Layer (interoperability), and the Security Layer (data privacy, encryption, authentication, and compliance with regulatory frameworks) [81]. The IoTWF Reference Model gained traction because of strong industry adoption as it provides a practical, vendor-neutral reference for IoT system design.

Later formal standardization efforts were reflected in the ISO/IEC 30141 IoT Reference Architecture [82,83], a standardized framework for designing and implementing IoT systems. The updated 2024 edition introduces improvements in usability, conformance, and implementation patterns. It defines a structured approach with six primary domains: the User Domain, Operations & Management Domain (OMD), Resource Access & Interchange Domain (RAID), Sensing and Controlling Domain (SCD), Physical Entity Domain (PED), and the cross-domain vertical functions of network connectivity, dynamic composition, and trustworthiness [83]. The inclusion of trustworthiness highlights the growing concern for cross-domain vertical functions such as network connectivity, dynamic composition, and human-in-the-loop. While sharing some conceptual similarities, IoTWF can be seen as a practical complement to the more abstract and standardized ISO/IEC 30141 framework.

#### 2.2.4. Off-the-Shelf IoT Architecture Implementations

Following up on the concepts established by the above-mentioned reference architectures, several concrete implementations tailored to particular use cases, environment, or organizations have emerged. One example is the AWS IoT, a cloud-based platform provided by Amazon Web Services that supports multiple communication protocols, including MQTT, HTTP, and WebSockets. One of its key features is the AWS IoT Greengrass service, which extends cloud capabilities to edge devices, enabling local data processing, machine learning inference, and offline operation. This is particularly important in industrial and healthcare IoT applications where low latency, real-time processing and regulatory compliance are critical [84,85].

Azure IoT enables secure, scalable, and AI-driven healthcare solutions by connecting medical devices, patient monitoring systems, and healthcare applications to the cloud. Through Azure IoT Hub, healthcare providers can securely collect, transmit, and analyze real-time patient data from wearable devices, remote monitoring tools, and hospital equipment, ensuring continuous health tracking and proactive intervention [86]. Azure IoT Edge extends cloud intelligence to on-premises medical environments, enabling real-time analytics for early disease detection, predictive maintenance of medical devices, and AI-assisted diagnostics without latency issues [87].

The Losant Enterprise IoT Platform offers the possibility of building IoT solutions with minimum coding, offering public cloud access under a free pricing scheme, and private cloud and on-premises installations for enterprise customers. The platform targets ease of use and deployment of IoT applications, by means of application templates that can be customized to fit specific scenarios such as smart hospital automation and AI-driven alerts for patients. The platform also supports edge computing to prefilter data to be sent to the cloud for processing and visualization [88,89].

The Particle Photon is a small, Wi-Fi-enabled IoT (Internet of Things) development kit that provides support to build connected products, including hardware, a cloud platform, and Arduino-style programming, making it easier to connect electronic components to the cloud. It has demonstrated value in connecting wearable health trackers and supporting IoT-enabled clinical trials [90].

Google also developed an IoT solution (Google Cloud IoT), which was discontinued in 2023 due to low adoption, market competition (in particular, with AWS IoT and Azure IoT) as well as shifting on their business strategy to focus on AI, data analytics, and cloud services [91]. In the same year, a similar decision was taken by IBM on their IBM Watson IoT [92].

Table 2 shows an analysis to these IoT architectures with a focus on their deployment model and how they align with the reference architectures.

#### 2.2.5. Gaps Identified

Despite the great advances in current systems, several critical gaps are still not completely covered for. One major limitation is data interoperability, as existing gateways struggle to integrate and harmonize information from diverse devices, protocols, and formats. This creates fragmentation and complicates data exchange between health systems. Additionally, current solutions often lack the ability to incorporate contextual and non-medical data—such as sleep patterns, activity levels, or environmental factors—which are essential for personalized and preventive healthcare.

Edge and fog computing capabilities are also underdeveloped. Many gateways rely too heavily on the cloud, leading to delays in data processing. A next-generation architecture should therefore support real-time analytics and decision-making closer to the data source, enabling local alert generation and low-latency machine learning inference. Security and privacy remain a persistent concern, especially with the sensitivity of health data. The architecture must support robust encryption, auditability, and potentially privacy-preserving AI techniques such as federated learning.

Scalability is also key, as the number of connected devices continues to grow. The architecture should be modular and efficient, both in hardware and software, to handle increasing loads without compromising performance.

Finally, existing gateways are often limited to simple data collection and transmission. A more advanced approach should enable support for digital twins, time-series analytics, and chronic disease monitoring directly at the edge or fog level, moving toward a more intelligent and autonomous healthcare infrastructure.

## 3. B-Health IoT Box

To address some of the identified gaps in current digital health infrastructures, we propose the development of the B-Health IoT Box, a modular and adaptable framework designed to bridge the gap between physical devices (such as sensors and medical instruments) and cloud-based applications [93]. The core innovation of the B-Health IoT Box lies in its fog computing architecture, which shifts key computing functions from remote cloud services to the edge of the network, closer to where data is generated. This approach supports responsiveness, bandwidth optimization, and regulatory compliance, especially in latency-sensitive or connectivity-constrained healthcare settings.

Functioning as an interoperable IoT hub, the B-Health IoT Box supports a wide range of devices regardless of manufacturer or communication protocol. It is capable of ingesting data in real time, transforming and storing it locally, applying intelligent processing pipelines, and forwarding relevant outputs to external systems. It also ensures compatibility with established eHealth standards (e.g., HL7 FHIR, openEHR [94]), facilitating integration into national or cross-border digital health infrastructures.

### 3.1. Architecture

**Figure 1:**
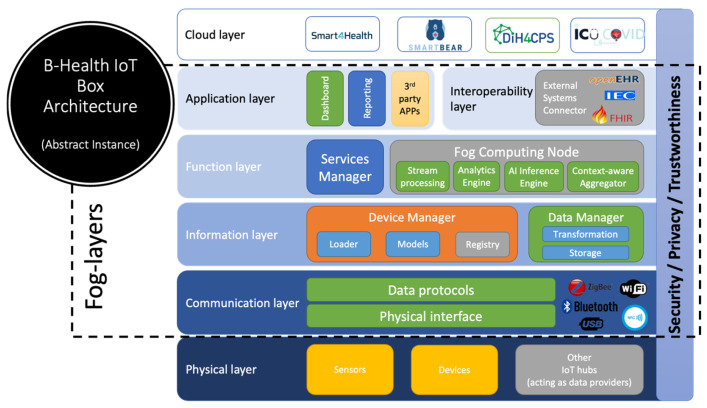
B-Health IoT Box architecture. Sensor data are acquired locally and processed within the fog layer, where harmonization (e.g., FHIR-based) and privacy-preserving preprocessing occur before transmission to the cloud.

The architecture of the B-Health IoT Box (Figure 1) follows the layered approach of the IoTWF Reference Model, structured across several interconnected layers to ensure modularity, scalability, and trustworthiness. This design enables seamless integration between physical devices, edge and fog computing capabilities, and cloud-based health applications, while each layer contributes with a specific role in ensuring seamless functionality and adaptability.

At the base, the physical layer allows for various health-related devices, including sensors, medical instruments, and other distributed IoT hubs that serve as data providers. Depending on the specific implementations, these components collect physiological and contextual information relevant to the healthcare domain.

Ensuring device connectivity using a combination of wired (e.g., USB) and wireless technologies (e.g., Bluetooth, Wi-Fi, Zigbee), the communication layer defines how data flows from the sensors to the IoT box. This layer abstracts the complexity of heterogeneous interfaces through support for widely used data protocols, enabling consistent and reliable data transmission. Building on the previous, the information layer handles the incoming data streams from sensors and devices, performing the necessary operations to make that data usable. Within this layer, a Device Manager identifies and processes the data model and properties of the connected devices, while a Data Manager interprets incoming data, applies basic transformations and filtering, and serves as a *queryable* storage component.

The function layer represents the core of the system, extending the computational capabilities of the IoT Box. The Service Manager is responsible for orchestrating the workloads assigned to the IoT Box and managing their lifecycle, including configuration, monitoring and maintenance tasks. The Fog Computing Node supports a range of use-case-specific tasks that enhance system responsiveness and local intelligence. It includes stream processing for managing continuous data flows and enabling real-time filtering and event detection; an analytics engine that performs lightweight statistical or rule-based analysis to extract insights directly; an AI inference engine capable of executing trained lightweight ML models locally to support predictive diagnostics and decision-making; and a context-aware aggregator that intelligently merges and prioritizes data based on relevance, urgency, or patient-specific conditions to optimize transmission and reduce unnecessary cloud interactions. Together, the analytics and AI inference engines provide the fog layer with an integrated capability for on-device intelligence. This combination enables local detection of abnormal physiological or environmental patterns and supports near-real-time responses without requiring cloud intervention. To extend these capabilities, the fog node architecture has been designed to accommodate federated-learning mechanisms, allowing distributed model training across multiple B-Health IoT Boxes while keeping sensitive data local. This privacy-preserving approach aligns with GDPR principles and supports scalable, intelligent edge computing in healthcare environments.

The application layer enables user interaction with the IoT Box. Through this layer, users can configure the system, visualize collected data through dashboards, or integrate third-party applications that rely on the underlying data and services. It also supports the development of custom applications that leverage the functionalities offered by the function layer, enabling, for example, notifications and AI suggestions. The interoperability layer, positioned at the same hierarchical level as the application layer, plays a critical role when integration with external systems or cloud platforms is required. While the application layer focuses on local interaction, configuration, and visualization, the interoperability layer enables connectivity with external health information systems and cloud-based infrastructures. It becomes especially relevant when cloud capabilities, such as remote analytics, storage, or integration with national or cross-border eHealth platforms, are needed. This layer supports established standards such as HL7 FHIR and openEHR and can be extended with custom connectors to meet specific interoperability requirements.

At the top, the cloud layer represents specific integrations with four EU H2020 cloud platforms, developed in the context of different projects, which are further discussed in Section 4 in the context of the B-Health IoT Box validation.

The fog-related components of the B-Health IoT Box are illustrated in Figure 1 within the dotted area, spanning from the communication layer up to the application and interoperability layers. These components collectively implement the system’s localized intelligence, data security, and responsiveness, distinguishing the B-Health IoT Box from conventional cloud-only healthcare architectures. This multi-layered, fog-enabled design ensures that the B-Health IoT Box can be deployed flexibly in various healthcare settings, from smart homes and clinical offices to intensive care units, facilitating timely insights, reducing cloud dependency, and ensuring robust interoperability with the broader digital health ecosystem.

### 3.2. Supported Standards and Protocols

To ensure seamless integration between physical health devices and cloud-based applications, the B-Health IoT Box leverages a combination of healthcare-specific standards and general-purpose communication protocols. This approach allows for a highly interoperable, modular, and future-proof solution tailored to the needs of connected health environments.

#### 3.2.1. Healthcare-Specific Standards

**Figure 2:**
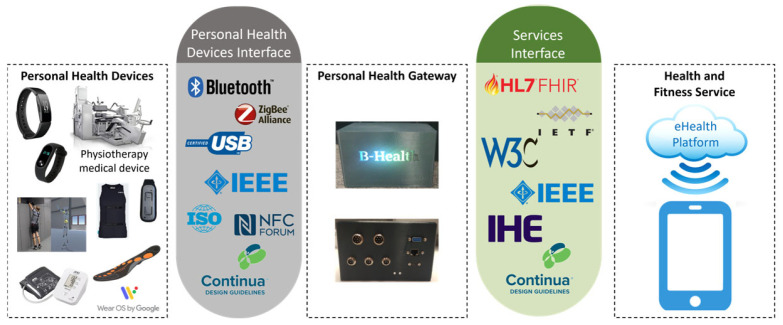
B-Health IoT Box CDG approach (adapted from [95]).

The B-Health IoT Box serves as a versatile data acquisition gateway, collecting information from a wide range of sources, including wearables, smart textiles, environmental and physiological sensors, and medical-grade devices. Conceptually aligned with the Personal Connected Health Alliance (PCHA) Continua Design Guidelines (CDGs), the B-Health IoT Box acts as a Personal Health Gateway (see Figure 2), ensuring a standards-based and interoperable architecture for health data integration.

Currently, the system natively supports data acquisition from various sources such as fitness trackers, smart t-shirts, smart insoles, posture monitoring sensors, CDG-compliant blood pressure monitors, and additional medical devices that adhere to common health communication protocols.

To extend compatibility beyond CDG-compliant devices, the platform incorporates custom adapters (wrappers), which were specifically developed to bridge non-compliant technologies with the CDG framework. These adapters were designed following the IEEE 11073 family of standards, enabling the semantic and structural alignment of device data. In addition, they accommodate the specific communication protocols and connectivity methods of the respective devices, including USB, Bluetooth Low Energy (BLE), Wi-Fi, and others. This extensibility ensures that the B-Health IoT Box can evolve alongside emerging technologies while maintaining robust interoperability and data consistency across heterogeneous health systems.

The B-Health IoT box is able to easily export collected data in FHIR. Generically, the B-Health IoT box can build and transmit any FHIR resource to a compatible FHIR server. By developing the appropriate connectors (at the interoperability layer), to accommodate authentication and/or data transformations for specific solutions, data stored or transiting through the B-Health IoT box can be transmitted, on-demand or automatically, to external entities.

The extensible nature of FHIR’s design allows the definition of resources that easily target different scenarios, while maintaining the basic operating premises. Connectors can be quickly written and/or adapted based on Profile specifications [96], and can even provide a more human readable approach using PDF embedded within FHIR resources [97].

By incorporating FHIR, the B-Health IoT Box can format and deliver patient data directly to Electronic Health Records and digital platforms using RESTful interfaces, ensuring both semantic and technical interoperability. The integration process is also aligned with the European Electronic Health Record Exchange Format.

#### 3.2.2. General-Purpose IoT Protocols and Interfaces

Complementing these healthcare standards, the B-Health IoT Box employs several general-purpose IoT protocols and interfaces to manage communication between devices and the cloud. JSON is used as the primary data format due to its simplicity and compatibility with modern APIs, including FHIR. For efficient, lightweight messaging, especially in bandwidth-constrained environments, the system uses MQTT, while HTTP/HTTPS provides secure web-based communication with remote services and cloud infrastructure.

To connect to a variety of physical devices, the IoT Box includes multiple hardware interfaces. Bluetooth Low Energy (BLE) enables short-range communication with mobile or wearable devices, while Zigbee supports low-power mesh networking in distributed sensor environments. Wi-Fi and Ethernet provide network connectivity, offering flexibility between wireless and wired deployments depending on the use case. Internally, the system uses I2C to interface with onboard sensors or embedded modules, facilitating custom hardware integration.

Finally, the B-Health IoT Box exposes RESTful APIs to external applications, enabling third-party systems to retrieve data, configure devices, or trigger workflows in real time. This API-driven architecture supports rapid integration into broader health IT ecosystems, research platforms, or cloud services.

Through this layered use of standards and protocols, combining healthcare-grade interoperability with general-purpose IoT connectivity, the B-Health IoT Box provides a robust foundation for building scalable, secure, and adaptable digital health solutions. Importantly, this list of supported standards and technologies is not exhaustive. The B-Health IoT Box was designed with flexibility and extensibility at its core, allowing it to incorporate additional protocols, data formats, and interoperability frameworks as new needs, technologies, or regulatory requirements emerge. This ensures that the platform can evolve alongside the rapidly changing landscape of connected healthcare. Future releases may include OPC UA support and 5G modules for enhanced throughput and security.

### 3.3. Fast-Prototyping Approach

The B-Health IoT Box was developed following a fast-prototyping methodology, enabling accelerated iteration and adaptation to evolve technical and functional requirements throughout the course of the research. This approach was particularly critical in the early stages of system design, where flexibility, modularity, and rapid feedback loops were prioritized over completeness or final form.

At the hardware level, the B-Health IoT Box was implemented using a Raspberry Pi 3 Model B+, a widely adopted single-board computer that features a 1.2 GHz quad-core ARM Cortex-A53 processor, 1 GB of RAM, built-in Wi-Fi and Bluetooth, and a range of I/O interfaces. The choice of this platform provided several advantages for prototyping: a low cost of entry, broad community support, compatibility with common sensors and modules, and sufficient processing power to support local data acquisition, lightweight processing, and communication with cloud services.

To accommodate the physical assembly and iterative development, the enclosure for the IoT Box was custom-designed and 3D-printed, leveraging additive manufacturing to speed up prototyping cycles. This enabled the team to rapidly adjust mechanical features such as port cutouts, sensor mounts, airflow openings, or LED indicators in response to emerging needs during testing and integration. The use of 3D printing also supported ergonomic and environmental constraints, such as wall mounting, field deployment, or access for maintenance, without the overhead of conventional enclosure manufacturing. Figure 2 includes an image of a 3D-printed B-Health IoT box in the centre.

From a systems architecture perspective, the B-Health IoT Box was engineered to support a Communication Layer capable of interfacing with a wide range of sensor types and data acquisition protocols. This includes digital and analogue sensors. Through this modular communication stack, the B-Health IoT Box supports real-time data acquisition and transmission from various sensing modalities, such as environmental parameters (temperature, humidity, CO_2_), physiological metrics (heart rate, SpO_2_), or contextual indicators (motion, light, sound). A key design principle was sensor extensibility: the system supports the dynamic addition of new sensors, both internal (mounted on the PCB or inside the enclosure) and external (connected via expansion ports or wireless interfaces). The plug-and-play capability is supported at both the hardware and software layers, with device abstraction to facilitate integration with minimal configuration. This allows the system to be easily reconfigured to meet new monitoring requirements as the use case evolves—for example, to track a different set of parameters, respond to a new health threat, or operate in a different physical environment. Note that the back panel of the 3D-printed box may be adjusted depending on the application scenario and the types of sensors to connect, especially if they require a wired-link.

Software-wise, the B-Health IoT Box includes drivers and communication modules that abstract low-level sensor interaction, exposing data through standardized APIs and message formats (e.g., JSON). These APIs integrate with the system’s IoT hub layer, which supports MQTT, HTTP(S), and RESTful services, and ultimately connects to cloud platforms or edge gateways for further processing, storage, and visualization.

Overall, the fast-prototyping approach is enabled by the use of open hardware (Raspberry Pi), additive manufacturing (3D printing), and modular, standards-based design practices, which significantly reduced the development cycle, increased adaptability, and ensured that the B-Health IoT Box could evolve in parallel with changing project goals. It also established a solid foundation for future productization, where prototypes can transition to more robust, industrial-grade platforms without discarding core architectural principles.

### 3.4. Data Collection Modes

The B-Health IoT Box was designed to support both real-time and near real-time data collection, adapting to the specific demands of various health monitoring and environmental sensing scenarios. These two operational modes are differentiated by the timing of data acquisition, processing, and transmission, and are enabled by the system’s modular architecture and flexible communication stack.

In real-time data collection, sensor readings are captured, processed, and made available for decision-making with minimal delay, typically within milliseconds. This mode is particularly relevant in time-critical applications, such as continuous physiological monitoring, where deviations in measurements may require immediate attention or trigger alerts. To facilitate this, the B-Health IoT Box relies on its computing architecture, performing localized processing to minimize latency and reduce the need for immediate cloud interaction. By using low-latency interfaces such as I2C and Bluetooth Low Energy, along with lightweight messaging protocols like MQTT, the system can stream sensor data in real-time to nearby processing units or dashboards. While it may not meet the stringent guarantees of hard real-time systems, the B-Health IoT Box effectively operates within the constraints of soft real-time environments, which are sufficient for most healthcare and wellness applications.

In contrast, near real-time data collection introduces a slight, controlled delay between the moment of acquisition and the moment of transmission or processing. This is often acceptable—and even advantageous—in use cases where immediate feedback is not required, but timely updates are still necessary. For instance, ambient environmental data such as temperature, humidity, or air quality may be collected at regular intervals and transmitted every few seconds or minutes. In these cases, the B-Health IoT Box buffers sensor readings locally, performs light preprocessing when needed, and transmits the data to cloud services or Electronic Health Record (EHR) systems. The use of standard formats like JSON and interfaces such as RESTful APIs ensures compatibility with health data platforms, including those based on FHIR.

By supporting both soft real-time and near real-time operations, the B-Health IoT Box offers a flexible foundation for a wide range of connected health scenarios, from acute monitoring and remote diagnostics to long-term wellness tracking and environmental health assessments, hence ensuring responsiveness, reliability, and adaptability in diverse deployment contexts.

In practice, the two data collection modes operate seamlessly within the same framework. In real-time operation, the B-Health IoT Box continuously acquires and processes sensor data locally, providing instantaneous visualization and alert capabilities when deviations are detected. In near real-time operation, data are stored temporarily within the device and periodically transmitted to external servers or health record systems using standardized formats and secure communication protocols. The coexistence of both modes allows the same infrastructure to be deployed in different contexts, such as clinical, workplace, or environmental scenarios, without altering the core configuration. This integrated design ensures efficient management of real-time signals and periodic measurements, while maintaining synchronization, interoperability and reliability across applications.

### 3.5. Security, Privacy and Trustworthiness

Given the intended use of the B-Health IoT box, security and privacy are key aspects. The system is built to support secure data flows from the physical layer to the cloud, aligning with general cybersecurity best practices and sector-specific requirements related to health data protection.

Device-Level Security—At the device level, the B-Health IoT Box operates on a secure Linux platform (e.g., Raspberry Pi OS or Debian) with configurable user privileges, access control, and system hardening. Secure Shell (SSH) access is enabled only through authorized key pairs, and unnecessary ports or services are disabled by default. Software updates and patches can be applied remotely or locally, ensuring that known vulnerabilities are mitigated in a timely manner. To further protect the integrity of the device, file system access can be restricted, and critical configuration files are monitored to prevent unauthorized modification. A firewall (e.g., iptables or nftables) is optionally deployed to filter network traffic and limit exposure to external threats.

Data Encryption and Communication Security—All data collected by the B-Health IoT Box can be encrypted at rest and in transit. Relying on the Linux standard LUKS (Linux Unified Key Setup) ensures that data stored locally (e.g., temporarily cached sensor readings) is stored encrypted. For data in transit, the system supports secure communication protocols such as HTTPS and MQTTS (MQTT over TLS). This ensures end-to-end encryption between the B-Health IoT Box and cloud services or external systems, safeguarding against eavesdropping and man-in-the-middle attacks. In environments where network reliability or external access is limited, data can be temporarily stored in encrypted buffers and transmitted securely when connectivity resumes.

Authentication and Access Control—To control system access and prevent unauthorized interactions, the B-Health IoT Box supports API-level authentication using tokens, credentials, or cryptographic keys. Access to administrative interfaces is password-protected and can be enhanced with multi-factor authentication (MFA) mechanisms when integrated with external identity management systems. At the software layer, individual modules and services within the B-Health IoT Box follow the principle of least privilege, minimizing the potential impact of compromised components or services. The system can also log access attempts, data transmission events, and security-relevant actions for audit and forensic purposes. These logs are maintained in secure storage with restricted access to authorized administrators only. Log data are retained for the period required by each deployment policy and are then safely deleted in accordance with applicable data-protection and ethical-research requirements. Each entry is time-stamped and linked to the corresponding process or user event to ensure traceability and accountability. The forensic workflow relies on these records to reconstruct sequences of operations, identify potential breaches, and verify the integrity of stored or transmitted data. This ensures that all logged information remains tamper-evident and that non-repudiation and access-control principles are effectively enforced throughout the system’s lifecycle.

Regulatory and Privacy-by-Design approach—The B-Health IoT Box prototype was developed following a privacy-by-design approach consistent with the principles of the European Union’s General Data Protection Regulation (GDPR). The prototype demonstrates how regulatory principles of data minimization, integrity, and accountability can be operationalized in an edge-computing healthcare environment. Sensitive data are pre-processed locally before transmission to reduce exposure, and all communication occurs through end-to-end TLS encryption with authenticated access control. To support auditability and traceability, data-handling operations and access attempts are recorded in tamper-evident encrypted logs. In addition, the architectural design supports the integration of privacy-preserving computation techniques, such as differential privacy and federated learning, enabling the exploration of distributed analytical models and statistical processing without exposing raw personal identifiers.

The described architecture ensures that data confidentiality, integrity and availability are preserved at every stage of operation. Security is embedded from device configuration to cloud communication through the combination of encrypted storage, authenticated connections and controlled user access. These measures establish a trustworthy and compliant framework that safeguards sensitive data throughout its entire lifecycle, from acquisition to long-term storage.

### 3.6. Contribution to Fill Existing Gaps

The B-Health IoT Box was conceived and developed precisely in response to several of the key limitations observed in existing healthcare IoT gateways. While mainstream platforms have evolved significantly, they often fall short in specific areas that are critical for modern, patient-centred, data-driven healthcare. The B-Health IoT Box takes a modular, standards-based, and flexible approach to mitigate the following identified gaps.

Interoperability of Devices, Protocols, and Formats—The B-Health IoT Box directly addresses this issue by supporting multiple communication protocols (e.g., MQTT, HTTP/HTTPS, Zigbee, BLE, I2C) and standardized data formats (e.g., JSON), and by integrating healthcare-specific standards such as FHIR and Continua Design Guidelines (CGD). This ensures that it can ingest and harmonize data from both certified medical devices and consumer health sensors, and output structured data that is compatible with Electronic Health Record (EHR) systems and cloud-based health platforms. As a result, it significantly reduces data fragmentation and enhances seamless data exchange across systems.

Integration of Contextual and Non-Medical Data—The B-Health IoT Box is designed to capture and integrate contextual data, such as physical activity, environmental conditions, and sleep patterns. Its modular sensor architecture and flexible communication layer allow easy addition of non-clinical sensors (e.g., accelerometers, air quality sensors, light sensors), making it suitable for personalized and preventive healthcare applications. This enriches the data context available for analysis and supports a more holistic view of the patient’s condition.

Edge and Fog Computing Capabilities—The B-Health IoT Box incorporates a fog computing architecture, which allows for on-device data processing, decision-making, and alert generation in real time. This enables the execution of basic analytics, threshold detection, and even local machine learning inference directly on the device. By reducing reliance on the cloud, the system ensures low-latency responsiveness, crucial for time-sensitive health interventions such as fall detection, vital sign monitoring, or anomaly detection.

Security and Privacy—The B-Health IoT Box was built with multi-layered security features. These include encryption at rest and in transit, SSH key-based authentication, configurable firewalls, and support for secure APIs. While it does not yet natively implement advanced privacy-preserving AI techniques such as federated learning, the system is architecturally ready to support such extensions by executing models locally and securely transmitting only processed outcomes. Additionally, its design supports auditability and access logging, which can be important in clinical trials or regulated environments.

Scalability and Modularity—The B-Health IoT Box supports scalability both in hardware and software. Its modular hardware design allows users to add or swap sensors and interfaces based on evolving requirements. On the software side, its microservice-inspired architecture and RESTful API integration facilitate interoperability with diverse systems and easy updates. This makes the platform adaptable to various deployment sizes—from small-scale home monitoring to larger research or institutional implementations—without sacrificing performance or maintainability.

Advanced Features—The B-Health IoT Box is designed to support local time-series analytics, basic trend detection, and future extensions such as digital twin modelling and edge-based chronic disease monitoring. Its fog computing core allows for persistent local data storage and retrospective analysis, supporting smarter decision-making at the point of data generation. While current implementations focus on foundational edge intelligence, the system architecture lays the groundwork for progressively incorporating predictive modelling, adaptive thresholding, and autonomous response mechanisms.

To contextualize the design and capabilities of the B-Health IoT Box, and despite the difference in levels of maturity scale, we provide a comparative overview against the above-mentioned Internet of Things (IoT) platforms, i.e., AWS IoT with Greengrass, Azure IoT with Hub and Edge, Losant IoT, and Particle Photon (Table 3). The comparison considers five key dimensions: (1) support for healthcare standards and interoperability frameworks; (2) the range and type of communication protocols supported; (3) capabilities in handling real-time and near real-time data collection; (4) ease of use in rapid prototyping scenarios; and (5) core security features at both the device and cloud levels.

In comparison to leading commercial IoT platforms, the B-Health IoT Box distinguishes itself through its native support for healthcare interoperability standards such as HL7 FHIR and the Continua Design Guidelines (CDG), its flexible fog-computing architecture, and its ability to operate in both real-time and near real-time data-collection modes. Unlike generic platforms that require additional adaptation layers to fit healthcare use cases, the B-Health IoT Box is designed from the ground up for connected-health IoT platforms applications, offering seamless integration with medical devices, modular sensor interfaces, and customizable communication protocols. Its use of open hardware (Raspberry Pi) and 3D-printed enclosures supports rapid prototyping and physical adaptability, allowing iterative customization, something less accessible in more rigid commercial ecosystems. Furthermore, its security-aware architecture enables local data processing, encrypted communication, and compliance with privacy regulations, making it a robust and adaptable platform for research, clinical monitoring, and environmental-health projects alike.

Beyond these practical features, the B-Health IoT Box also introduces methodological innovations that differentiate it from existing fog- or edge-based IoMT architectures such as AWS Greengrass, Azure IoT Edge, and openEHR-based gateways. Whereas most commercial systems remain cloud-centric and depend on proprietary integration workflows, the B-Health IoT Box embeds semantic harmonization directly within the fog layer through the combined use of HL7 FHIR and CDG-compliant data models. This approach transforms the fog gateway from a simple relay into an active semantic-processing node, capable of performing privacy-preserving preprocessing and real-time data normalization at the edge. Consequently, the system reduces latency, lowers bandwidth consumption, and ensures consistent interoperability across heterogeneous devices and platforms. Moreover, the modular design of the B-Health IoT Box allows the same architecture to operate across diverse deployment contexts, from clinical to industrial (as shown in Section 4), without reconfiguration, demonstrating its potential as a reusable architectural blueprint for future interoperable eHealth ecosystems.

## 4. Application Scenarios

The B-Health IoT Box was designed to facilitate the addition of more sensors (both internal and external), enabling to plug-and-play integration of different kinds of sensors through its communication layer, according to new requirements and parameters to be monitored. This versatility allows it to be used in different scenarios where the main focus is data collection, harmonization, and data transfer using eHealth standards. It can be seamlessly reconfigured for clinical, home, occupational, or community environments, supporting a wide range of health and wellbeing services.

Experimental deployments have followed ethical approval procedures and compliance with the principles of the General Data Protection Regulation (GDPR), guaranteeing lawful data processing and informed consent from participants. Privacy is maintained by limiting data exposure to authorized users and by ensuring that collected information is anonymized or pseudonymized according to applicable regulations.

The following subsections present different scenarios where the B-Health IoT Box is used and validated. In all of them, a co-creation approach was used with the B-Health IoT Box users for the requirements elicitation. This enables a person-centred development, ensuring that the B-Health IoT Box fits the users’ needs.

### 4.1. Collection of Back Pain Prevention and Treatment Training Data

This scenario was tested and validated in the scope of two Horizon 2020 European projects: (i) the Smart4Health project (www.smart4health.eu), aiming at empowering European Union (EU) citizens with an interoperable and exchangeable Electronic Health Record (EHR) that allows EU citizens to be active participants in managing their own health information; and (ii) the Smart Bear project (www.smart-bear.eu), delivering a solution that offers measurable improvement to the Quality of Life of the elderly and their ability to live independently, while also contributing to a continuous and objective monitoring with personalized interventions towards optimizing disease and associated risk management.

**Figure 3:**
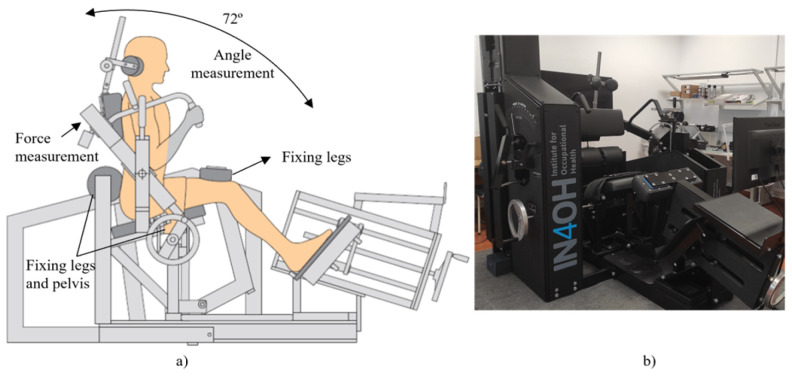
(**a**) MedX LE physiotherapy machine schema. (**b**) MedX LE physiotherapy machine picture.

In this regard, both projects foresee the integration of data coming for various devices, including a class IIa medical device, the MedX Lumbar Extension (LE) physiotherapy machine (Figure 3). The MedX LE device is designed to isolate and strengthen the muscle groups in the low back that support the lumbar spine and has the following as its main characteristics: fixation for legs and pelvis, isolation of lumbar extensors, balance of upper body weight in gravity with a counterweight, range of motion of 72°, and a weight stack from 1 to 400 pounds (0.5 to 200 Kg).

The lumbar extension training protocol is a digitally supported physiotherapeutic procedure designed to evaluate and strengthen the lumbar musculature through a structured sequence of assessments and exercises. Following each training session, a FHIR DiagnosticReport resource is automatically generated, encapsulating the key metrics from that session. This includes the training score, number of repetitions, weight applied, session duration, and calories expended. This standardized resource ensures semantic interoperability with electronic health records and other health information systems. The physiotherapeutic process supported by the system encompasses three primary procedures:

1. Assessment of the person’s range of motion to evaluate flexibility and joint limits.
2. Force testing of the lumbar region, which quantitatively measures muscle strength with a precision of 3° increments across the lumbar range.
3. Resistance training, where the patient performs controlled exercises across the full range of lumbar motion under adjustable load conditions.

These procedures are conducted using a MedX training machine, which is equipped with a native sensor suite comprising the following:

- A switch button used by the physiotherapist to interact with the system, marking events such as exercise initiation, termination, or intermediate evaluation points during force tests.
- An angle sensor, implemented as a potentiometer, that provides real-time angular position readings. These are derived from electrical resistance variations, linearly correlated to the degree of lumbar flexion or extension.
- A force sensor, based on a load cell, that captures the dynamic force exerted by the patient throughout each phase of motion, enabling both strength assessment and biofeedback.

To enhance the precision and safety of training, and to support real-time corrective feedback, the system has been extended with additional body-positioning sensors. These are strategically placed at the head, back, legs, and feet of the user [93]. Their function is to monitor body alignment and positioning during exercise execution, providing instantaneous feedback to both the patient and the physiotherapist. This not only helps ensure biomechanical correctness but also improves the effectiveness of the training by reducing compensation patterns and injury risk.

**Figure 4:**
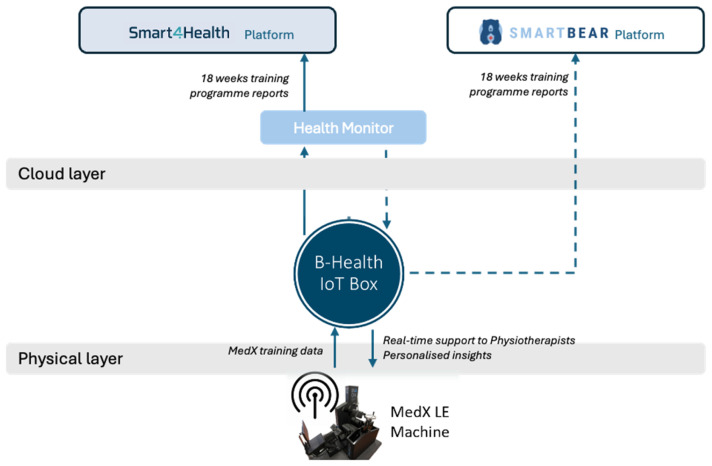
Data collection architecture for back pain prevention and treatment training data.

The B-Health IoT Box connects all sensors’ data at the physical layer, with wired cables, and at the data collection layer, with the interfaces made available from the Smart4Health (via SDK) and SmartBear cloud platforms (via REpresentational State Transfer (REST) Application Programming Interfaces (APIs)). Besides performing data collection, the B-Health IoT Box is responsible for cleaning and aggregating data, providing it in a FHIR format to HealthMonitor, a software for physiotherapists to manage the Lower Back Pain (LBP) physiotherapy programme. Due to different architectural decisions at each project, the way the results from the programme reach the platforms is different (Figure 4). In the case of Smart4Health, the MedX training data collected during the physiotherapy session is processed by the HealthMonitor and transferred to the Smart4Health platform via a specific Smart4Health connector, used to ensure interoperability and secure data transfer between the two systems. The connector requires that a successful pairing is established between the systems before the upload. In the case of Smart Bear, the Health Monitor returns the processed training data to the B-Health IoT Box which is then responsible for sending it to the Smart Bear platform using a dedicated pairing service which was, in this case, established at the Box.

**Figure 5:**
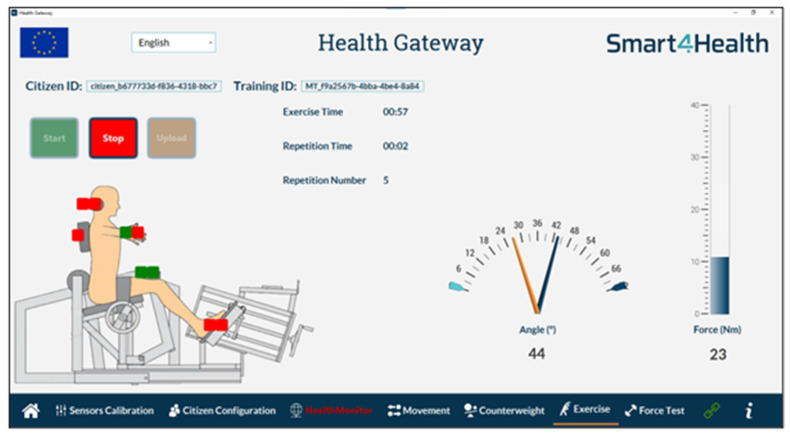
Health Gateway application interface.

In addition to this, the Health Gateway application was deployed at the B-Health IoT Box application layer to support the person during the physiotherapy exercise (Figure 5). The Health Gateway provides real-time feedback on the accuracy of the exercise allowing the correction of speed and position. Having this interaction has proved to be beneficial to keep motivation during the exercise and contribute to a successful accomplishment of the 18-week LBP programme.

Overall, this integrated sensor-based framework, combined with FHIR-based clinical documentation, offers a comprehensive and interoperable solution for lumbar physiotherapy—enabling data-driven assessment, personalized feedback, and clinical traceability within modern health information ecosystems.

### 4.2. Collection of Environmental, Health and Wellbeing Data at Work

**Figure 6:**
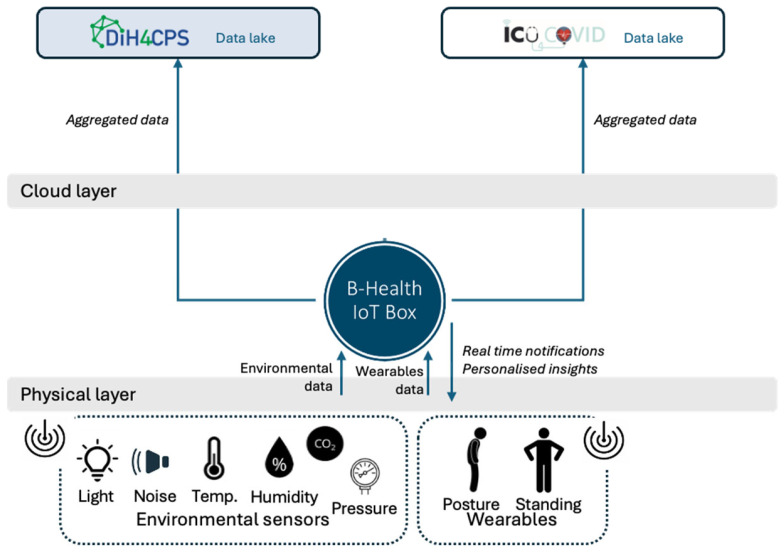
Data Collection architecture for wearables and environmental sensors.

This scenario was tested and validated in the scope two Horizon 2020 European projects (i) DIH4CPS (https://www.hipeac.net/network/projects/7125/dih4cps/#/ (accessed on18 September 2025)) aiming to target workers suffering from different work-related types of physiological and posture-related issues and (ii) ICU4COVID aiming to deliver intensive care medicine fit for the fight against COVID-19 (Figure 6). In addition, the testing of the scenario is being evaluated in the context of Horizon Europe project AGILEHAND (www.agilehand.eu (accessed on18 September 2025)) which aims at developing advanced technologies for grading, handling and packaging autonomously soft and deformable products.

The main objective of the B-Health IoT Box within this scenario is to monitor workers and their environment (both industrial and ICU staff), identifying potential issues that can lead to long-term health problems. In this regard, data form environmental sensors (i.e., CO_2_, temperature, humidity, luminosity) together with data from workers’ posture are collected by the B-Health IoT Box and made internally available for the authorized personnel. For this purpose, appropriate smart wearables were deployed to monitor the consenting workers while carrying out their work activities. The measured parameters include standing hours and posture. Also, the workplace was equipped with smart devices to collect information about the work environment.

In the case of DIH4CPS, two B-Health IoT Boxes were deployed at three different settings, i.e., shop floors. Each deployed box was responsible for monitoring the environmental conditions of a specific working station and its worker. In the case of ICU4Covid, four B-Health IoT boxes were deployed in four different settings, i.e., hospitals. Each deployed box was responsible for monitoring one ICU unit together with the healthcare staff using the wearables. To differentiate between the staff, a log system was created. In all cases, all raw data was transferred (either in real-time or batch) wirelessly, e.g., Bluetooth Low Energy (BLE), to the B-Health IoT Box.

**Figure 7:**
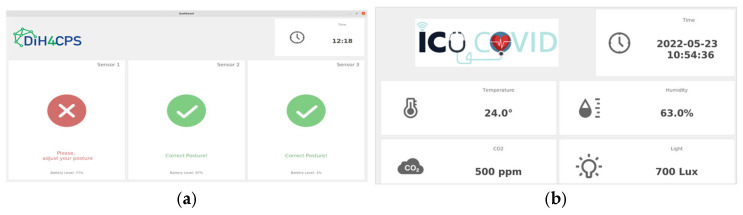
(**a**) B-Health IoT Box posture dashboard on a tablet. (**b**) B-Health IoT Box Environmental dashboard.

In the B-Health IoT Box, an app was deployed at the application layer to interpret data and translate it to activities like “sitting with good or bad posture” so that corrective actions are provided to the worker as feedback, to support their properly reaction (e.g., take a break to rest, use the strength of their legs to lift heavy packages). To notify the worker, a specific dashboard was deployed in a tablet placed at the workstation to demonstrate the position that should be adopted to maintain good posture (Figure 7a). This tablet was only used in DIH4CPS as it was seen as something to not be used in the ICU. In this case, healthcare staff could log into to the system to check their measures. In addition, a dashboard was developed in the B-Health IoT Box to visualize environmental information in real time (Figure 7b). All information collected is then processed to determine optimal and suboptimal working conditions.

This comprehensive setup demonstrates how the B-Health IoT Box, integrated with smart wearables and environmental sensors, enables the real-time monitoring of workers and working conditions, providing actionable feedback and standardized data reporting, thereby contributing to safer and more ergonomic work environments. Also, metrics collected are reported using a FHIR observation resource capable of being integrated in PHRs. These real-world evaluations confirmed the effectiveness of the B-Health IoT Box in supporting remote monitoring, personalized care, and digital health innovation.

## 5. Validation and Discussion

The validation activities conducted for the B-Health IoT Box demonstrate a comprehensive assessment of its performance, usability, and integration across multiple operational domains. The system was evaluated in the context of four Horizon 2020 projects—Smart4Health, SmartBear, DIH4CPS, and ICU4Covid—each representing distinct environments including physiotherapy practices and industry and healthcare workplaces. This multi-context validation highlights the B-Health IoT Box’s adaptability and reliability in diverse health-related scenarios.

A key strength of the B-Health IoT Box is its compliance with international health data standards, particularly HL7 FHIR. This ensures full and easy interoperability alignment with major European initiatives, including the future European Health data Spaces, as well as enables structured and meaningful data exchange.

In the collection of back pain prevention and treatment training data scenario, more than 80,000 datasets related to MedX training were collected and ingested in the project’s platforms through the B-Health IoT Box. These datasets contain information from more than 4500 citizens and patients that engaged in the projects and performed the full 18-week training as well as from those that only did the assessment and force test.

In the collection of environmental, health and wellbeing data at work scenario, more than 1.5 million datasets related with environmental conditions at work as well as to worker posture were collected ingested in the project’s data lakes through the B-Health IoT Box. The B-Health IoT Boxes were used during 1-month, at each site, monitoring 5 workers (in the case of DIH4CPS) and 25 healthcare professionals (in the case of ICU4Covid) during their shifts. Table 4 summarizes key metrics across the different use cases scenarios.

As most real-world applications, some issues were discovered during the on-site deployment and operation. Due to limited wireless range of the B-Health IoT Box, its placement was crucial to ensure a smooth and uninterrupted data collection. A network of B-Health IoT Boxes was envisaged to address this issue in larger deployment sites. The system was able to ensure consistent data acquisition rates and low latency in processing and transmission.

To quantitatively assess system performance and reproducibility, a controlled validation was conducted in the physiotherapy setting under laboratory conditions. During a three-minute MedX training session, 1785 samples were collected to measure key metrics including latency, throughput, and payload efficiency. The B-Health IoT Box achieved an average end-to-end latency of 6 ms (minimum 1 ms; maximum 58 ms) for the complete processing chain, i.e., sensor reading, FHIR message construction, data transmission, and visualization. Throughput reached up to 80 observations per second when transmitting batched FHIR messages (10 samples per array), confirming the system’s ability to sustain real-time feedback and low-latency operation. The methodology followed in this validation replicated the operational setup used in the physiotherapy scenario, ensuring that the measurements reflected realistic conditions of use within the MedX equipment. All tests were performed using the native configuration of the B-Health IoT Box and its embedded software modules, connected to the MedX sensors and interface without any modification to hardware or sampling frequency. The recorded timings therefore represent the actual end-to-end performance of the system in its production configuration. Additional validation runs were performed in the same environment to verify the repeatability of the results and the consistency of the data-collection process. In all cases, the B-Health IoT Box maintained stable acquisition rates and similar latency values, confirming the robustness of the architecture and the reliability of the measurements. These repeated tests, together with the successful operation of the system in multiple pilot deployments described earlier, demonstrate that the performance observed in this validation scenario is representative of the platform’s overall behaviour under real-world conditions.

To further evaluate payload efficiency, two transmission tests were conducted: (1) the data samples were transmitted individually in single FHIR observations, and (2) the data samples were sent in array of 10 observations. Table 5 presents the comparative results obtained in both tests.

Table 4 and Table 5 together provide a quantitative and qualitative view of the framework’s performance and scalability. Table 4 demonstrates the breadth of operational contexts and data volumes successfully supported by the B-Health IoT Box, from more than 80,000 datasets in physiotherapy training to over 1 million datasets in hospital-workplace monitoring. These values confirm that the same modular platform can handle both small-scale clinical sessions and large, continuous workplace deployments without structural modification. Table 5 complements these findings by quantifying communication and processing efficiency. The results show that sending observations in batches of 10 reduces total transmission time from ~79 ms to ~12 ms for comparable workloads, i.e., a performance gain of roughly 6×, while increasing payload volume by only ~21%. This demonstrates the capability of the system to balance latency and bandwidth, offering real-time local responsiveness when required and near-real-time efficiency when scalability and throughput are prioritized. Together, the two tables illustrate that the B-Health IoT Box delivers real-time, low-latency processing and robust, standards-compliant large-scale data ingestion, providing tangible added value compared with conventional IoT health solutions.

In contrast with existing solutions in the market, which are frequently vendor-locked, the B-Health IoT Box leverages open-source platforms and FHIR-based data exchange, offering healthcare-native interoperability that is not available out-of-the-box in AWS or Azure. The measured response times of 6 ms for local feedback is particularly relevant in healthcare, where feedback must be timely and reliable to be clinically effective (e.g., posture correction, temperature alerts, or ergonomic warnings). Also, and although the experimental conditions and scale differ, a comparison between the B-Health IoT Box and available benchmark results [98] for Microsoft Azure IoT Edge and AWS Greengrass was established. While a direct numerical comparison is difficult, since the test setups differ in workload complexity, network environment, and hardware configuration, the benchmark provides a useful reference point, indicating that the B-Health IoT Box operates within the expected performance range of contemporary edge-computing platforms, while also integrating a FHIR-compliant framework for healthcare data exchange.

Another differentiating factor is the B-Health IoT Box’s hardware flexibility and open prototyping model. Built around the Raspberry Pi architecture, and supported by 3D-printed enclosures, it allows rapid adaptation to specific sensor types and physical constraints. Commercial platforms often require hardware customization or are tied to fixed device configurations, making them less suitable for research-oriented or domain-specific experimentation. Across all pilots, data was encrypted during transmission and persisted securely in the cloud, following GDPR-compliant protocols.

From a data perspective, the validation suggests that the B-Health IoT Box effectively manages heterogeneous data sources across all application scenarios. The same standards-based pipeline handles both real time signals from MedX training sessions and periodic environmental and posture data from workplace settings. Collected information can be converted into HL7 FHIR-compliant structures and transmitted to compliant external HL7 FHIR servers. The results obtained, that are ranging from the 6 ms local processing cycles in physiotherapy tests to the multi-million-dataset ingestion in workplace monitoring, show that the data architecture supports both immediate feedback and large-scale analytics in a unified manner.

Each deployment operated under encrypted communication channels, secure data storage and controlled user authentication, maintaining the integrity and confidentiality of sensitive information throughout the experiments. All pilots were conducted under approved ethical procedures and in conformity with GDPR principles, guaranteeing lawful processing, anonymization and informed consent for data usage. This integration of robust data-management and security safeguards ensures that the B-Health IoT Box can be reliably deployed in real-world healthcare and workplace environments while upholding both technical and ethical standards.

Overall, the results of the pilot deployments and laboratory evaluations validate the B-Health IoT Box as a high-performance, interoperable and versatile solution for health and wellbeing monitoring. Its architecture enables scalable deployment and future extension, aligning with the evolving needs of digital health ecosystems.

## 6. Conclusions and Future Work

This paper presents the design, implementation, and validation of the B-Health IoT Box, a modular, standards-compliant gateway for edge and fog-based health data collection. Drawing upon deployments in four major Horizon 2020 projects, Smart4Health, SmartBear, DIH4CPS, ICU4Covid, and AGILEHAND, the study demonstrates the adaptability of the B-Health IoT Box across diverse settings, including physiotherapy, working environment and ergonomics. The system collected millions of data points from over 4500 users, across more than 15 pilot sites in real-world healthcare and workplace contexts.

The results provide strong evidence of the system’s performance, particularly in terms of latency, data integrity, and real-time feedback capabilities. The architecture supports plug-and-play sensor integration and complies with health data standards such as HL7 FHIR, enabling seamless interoperability with various cloud platforms. This standards-based approach is a critical strength, addressing one of the most pressing challenges in the digital health ecosystem: data interoperability across heterogeneous systems. In comparative benchmarking, the B-Health IoT Box outperformed typical commercial cloud-based IoT platforms in latency, adaptability, and healthcare-specific interoperability.

Beyond performance metrics, the B-Health IoT Box showcases how fog computing can mitigate the limitations of cloud-reliant architectures. By processing data closer to the source, the system reduces network congestion and supports immediate feedback loops, essential in use cases like physiotherapy and posture correction. This decentralized approach also improves resilience, as buffering mechanisms ensured continuity during temporary connectivity loss. Notably, the system achieved sub-10ms responsiveness in real-time training settings. However, the study also revealed opportunities for future enhancements. These include systematic failure logging, broader comparison with other commercial systems, and integration of user-centric design evaluation. Security and privacy practices were enforced in line with GDPR, but future work could investigate privacy-preserving machine learning techniques, such as federated learning, to further strengthen compliance.

The evidence gathered across the validation activities confirms that the proposed framework achieves high performance, scalability, and reliability while maintaining strict compliance with data-protection and interoperability standards. The system sustained continuous multi-sensor data acquisition across heterogeneous environments, combining low-latency edge responsiveness with efficient large-scale data transmission. These results demonstrate that the B-Health IoT Box provides a practical and secure solution for real-time and near-real-time digital-health applications, ensuring both technical excellence and regulatory trustworthiness. Its open-source prototyping model, real-world performance validation, and strong adherence to healthcare standards position it as a scalable and future-ready solution for digital health innovation.

Future directions include expanding deployment to national healthcare systems, incorporating AI-driven local inference for chronic disease detection, and strengthening cloud-to-edge integration with automated configuration and diagnostics. Furthermore, the integration of AI-driven graph or hypergraph-based inference models [99], represents a promising direction for enabling multi-source data fusion and advanced predictive interoperability across heterogeneous IoMT ecosystems.

## Figures and Tables

**Table 1:** Common protocols for IoMT interoperability.

	Protocol	Interfaces
Data formats	JSON **^1^**	Lightweight, human-readable format
	XML ^2^	Structured, verbose; used where schema definition is important
	CBOR ^3^	Efficient binary format for constrained devices
Communication protocols	MQTT ^4^	Lightweight pub/sub protocol ideal for low-bandwidth IoT scenarios.
	HTTP/HTTPS ^5^	Standard web protocol for IoT dashboards and RESTful services.
	COAP ^6^	Web transfer protocol optimized for constrained devices and networks.
	Zigbee ^7^	Low-power mesh networking protocol
Interfaces	Bluetooth/BLE ^8^	Short-range wireless interface/Bluetooth Low Energy
	Wi-Fi ^9^	Standard wireless networking interface for many IoT devices
	RESTful APIs ^10^	Standardized interface for interacting with IoT devices and services over HTTP
	Ethernet ^11^	Wired interface, reliable for industrial and local IoT setups.
	I2C ^12^	Short-distance communication interface used to connect sensors and peripherals in IoT

^1^ https://www.json.org/. ^2^ https://www.w3.org/TR/xml/. ^3^ https://cbor.io/spec.html. ^4^ https://mqtt.org. ^5^ https://datatracker.ietf.org/doc/html/rfc2616; https://datatracker.ietf.org/doc/html/rfc2818. ^6^ https://datatracker.ietf.org/doc/html/rfc7252. ^7^ https://csa-iot.org/all-solutions/zigbee/. ^8^ https://www.bluetooth.com. ^9^ https://www.ieee802.org/11/. ^10^ https://fetch.spec.whatwg.org. ^11^ https://www.ieee802.org/3/. ^12^ https://www.i2c-bus.org/, all accessed on 18 September 2025.

**Table 2:** Analysis of off-the-shelf architecture implementations.

Architecture	Fit in ISO/IEC 30141	Fit in IoT World Forum	Deployment Model
AWS IoT (+AWS Greengrass)	-Fits into the Functional View and System Deployment View -Supports cloud-based and edge-based processing (+AWS Greengrass)	-Covers the Application Layer (data insights) and Business Layer (data-driven decisions) -Can extend to the Data Processing Layer (+AWS Greengrass)	Primarily cloud-based but supports hybrid edge + cloud (+AWS Greengrass)
Azure IoT (+Hub) (+Edge)	-Covers the Functional View and Usage View -Part of the Networking View and Functional View (+Hub) -Maps to the System Deployment View and Functional View (+Edge) -Local data processing, containerized workloads, and AI at the edge (+Edge)	-Spans across the Application Layer, Business Layer, and Management Layer -Sits within the Network Layer and Management Layer (+Hub) -Falls into the Data Processing Layer and partially the Management Layer (device control and monitoring) (+Edge)	Primarily cloud-based but supports Edge deployment model with cloud synchronization (+Edge)
Losant IoT	Aligns mainly with Service Layer (application logic, orchestration) and Application Layer (user interaction, visualization)	Predominantly in the Application Layer, Business Layer, and Data Abstraction Layer	Cloud-native platform (SaaS) for IoT application development and orchestration
Particle Photon IoT	Fits in the Device Layer and partially the Network Layer	Primarily maps to the Edge Devices Layer and Network Layer, with some support for Data Abstraction	Device-centric with Particle Cloud for connectivity, OTA updates, and basic data management

**Table 3:** Comparative overview of IoT platform features.

Feature	B-Health IoT Box	AWS IoT + Greengrass	Azure IoT + Hub/Edge	Losant IoT	Particle Photon
Healthcare Standards	FHIR, CDG (Continua), healthcare-focused	No native support (FHIR integration requires customization)	FHIR support via Azure API for FHIR and Microsoft Health Cloud	No native healthcare standards	No support for FHIR/CDG
IoT protocols Supported	MQTT, HTTP/HTTPS, Zigbee, BLE, I2C, REST APIs, etc.	MQTT, MQTT over WebSockets, HTTPS, LoRaWAN, custom via Greengrass	MQTT, AMQP, HTTPS, custom via Edge runtime	MQTT, REST, WebSockets, custom parsing	MQTT, TCP/UDP, proprietary Particle protocol, REST API
Data collection modes	Real-time and near real-time; supports fog computing (edge)	Real-time + batch (Edge + Cloud); Greengrass enables local actions	Real-time + scheduled; strong cloud sync with Edge runtime	Near real-time focus; supports rules and edge logic	Real-time telemetry, constrained by hardware
Fast prototyping support	Raspberry Pi + 3D printing + modular sensor architecture	High barrier for device setup; great for scalable systems	Requires Azure setup; powerful but complex for quick testing	Strong visual workflow and drag-and-drop prototyping	Hardware-focused prototyping (dev boards, cloud integration)
Security features	SSH access, local encryption, customizable security stack	Strong IAM, encryption, policy-based access, secure device certs	Azure Active Directory, per-device auth, encryption, TPM support	Built-in auth, role-based access, SSL	Secure cloud access, device keys, firmware signing

**Table 4:** Key metrics across the different use case scenarios.

Scenario	Types of Users	N° of Users	N° of Deployed B-Health IoT Boxes	N° of Sensors (Per Box)	N° of Datasets Collected
Collection of back pain prevention and treatment training data	Patients, physiotherapists	>4500	12 (1/MedX)	12	>80,000
Collection of health and wellbeing data at work	Industry workers	5	2 (1/working station + worker)	6 environ. + 2 wearables	>570,000
Collection of environmental data at work	Health professionals	25	4 (1/ICU unit + ~6 healthcare professionals)	6 environ. + 12 wearables	>1,000,000

**Table 5:** Comparative results obtained in tests.

	Test 1: Individual Observation		Test 2: Batch of 10 Observations	
**Nº of samples**	1000	10,000	100	1000
**Nº of observations**	1000	10,000	1000	10,000
**Average time per observation (ms)**	79	77	12.7	12.2
**Average time for cloud answer (ms)**	79	77	127	122
**Total test time (ms)**	79,499	769,072	12,788	122,654
**Size per FHIR message (B)**	345	345	4193	4193
**Total data sent (KB)**	345	3450	419.3	4193

## Data Availability

Data collected is unavailable due to privacy and ethical restrictions.

## Abbreviations

The following abbreviations are used in this manuscript:

3D	Three dimensional
AGILEHAND	Smart Grading, Handling and Packaging Solutions for Soft and Deformable Products in Agile and Reconfigurable Lines
AI	Artificial Intelligence
API	Application Programming Interfaces
AWS	Amazon Web Services
BLE	Bluetooth Low Energy
CDG	Continua Design Guidelines
CoAP	Constrained Application Protocol
DICOM	Digital Imaging and Communications in Medicine
DIH4CPS	Fostering DIHs for Embedding Interoperability in Cyber-Physical Systems of European SMEs
EC	European Commission
ECG	Electrocardiogram
EHR	Electronic Health Record
EU	European Union
(SMART on) FHIR	(Substitutable Medical Applications and Reusable Technologies on) Fast Healthcare Interoperability Resources
GDPR	General Data Protection Regulation
GHz	Giga Hertz
H2020	Horizon 2020
HDP	Health Device Profile
HE	Horizon Europe
HIPAA	Health Insurance Portability and Accountability Act
HIS	Health Information System
HL7	Health Level Seven
HTTP(S)	Hypertext Transfer Protocol (Secure)
I2C	Inter-Integrated Circuit
I/O	Input/Output
ICU	Intensive Care Unit
ICU4COVID	Cyber-Physical Intensive Care Medical System for COVID-19
IEC	International Electrotechnical Commission
IEEE	Institute of Electrical and Electronics Engineers
IG	Implementation Guide
IHE	Integrating the Healthcare Enterprise
IoT	Internet of Things
IoMT	Internet of Medical Things
IoTWF	Internet of Things World Forum
ISO	International Standards Organization
IT	Information Technologies
LED	Light-Emitting Diode
LUKS	Linux Unified Key Setup
(K)B	(Kilo) Byte
M2M	Machine to Machine
mCSD	Mobile Care Services Discovery
MedX LE	MedX Lumbar Extension
MFA	Multi-Factor Authentication
ML	Machine Learning
MQTT	Message Queuing Telemetry Transport
OMD	Operations & Management Domain
OPC UA	Open Platform Communications—Unified Architecture
OS	Operating System
OTA	Over-The-Air
PCB	Printed Circuit Board
PCD	Patient Care Device
PCHA	Personal Connected Health Alliance
PDF	Portable Document Format
PED	Physical Entity Domain
PHDC	Personal Health Device Class
PHR	Personal Health Record
RAID	Resource Access & Interchange Domain
REST	REpresentational State Transfer
SaaS	Software as a Service
SCD	Sensing and Controlling Domain
SDK	Software Development Kit
Smart4Health	Citizen-centred EU-EHR exchange for personalized health
Smart Bear	Smart Big Data Platform to Offer Evidence-based Personalized Support for Healthy and Independent Living at Home
SSH	Secure Shell
TLS	Transport Layer Security
WHO	World Health Organization
XDS	Cross Enterprise Document Sharing
XML	Extensible Markup Language
